# Effects of Seven Weeks of Combined Physical Training on High-Density Lipoprotein Functionality in Overweight/Obese Subjects

**DOI:** 10.3390/metabo13101068

**Published:** 2023-10-10

**Authors:** Tiziana Bacchetti, Camilla Morresi, Gianna Ferretti, Anders Larsson, Torbjörn Åkerfeldt, Michael Svensson

**Affiliations:** 1 Department of Life and Environmental Sciences, Marche Polytechnic University, Via Brecce Bianche, 60131 Ancona, Italy; c.morresi@staff.univpm.it; 2 Department of Clinical Science and Odontostomatology, Marche Polytechnic University, Via Brecce Bianche, 60131 Ancona, Italy; g.ferretti@staff.univpm.it; 3 Center for Health Promotion, Marche Polytechnic University, Via Brecce Bianche, 60131 Ancona, Italy; 4 Department of Medical Sciences, Clinical Chemistry, Uppsala University, 751 85 Uppsala, Sweden; anders.larsson@akademiska.se (A.L.); torbjorn.akerfeldt@akademiska.se (T.Å.); 5 Section of Sports Medicine, Department of Community Medicine and Rehabilitation, Umeå University, 90 187 Umeå, Sweden; michael.svensson@umu.se; 6 Umeå School of Sport Sciences, Umeå University, 90 187 Umeå, Sweden

**Keywords:** lipoproteins, oxidative stress, obesity, overweight, physical exercise

## Abstract

Our study aimed to investigate the effects of exercise on HDL composition and functional properties in overweight/obese subjects. Eighteen overweight/obese subjects (nine F and nine M, BMI = 30.3 ± 3 kg/m^2^) attended supervised training for 7 weeks. The protocol included combined resistance and conditioning training four to five times each week. The activity of the antioxidant enzyme paraoxonase-1 (PON1) associated with HDL was evaluated in all subjects before and after the training intervention. Moreover, myeloperoxidase (MPO) levels and oxidative stress markers (ox-LDLs and total antioxidant capacity) were studied in the serums of the subjects. At the end of the intervention, the activity of PON1 was increased (*p* < 0.0001), and MPO levels and the MPO/PON1 ratio were decreased (*p* < 0.0001). In addition, a significant improvement in muscle strength and maximal oxygen uptake (VO_2_max) (*p* < 0.0001) and a significant reduction in total and visceral adipose tissue mass (*p* < 0.001) and waist circumference (*p* < 0.008), without any significant decrease in body weight, were observed. A significant correlation was established between serum MPO/PON ratios, HDL redox activity and ox-LDLs. In conclusion, our results demonstrate that exercise training, without modifications of dietary habits, improved HDL functionality in overweight/obese adults, without any significant reduction in BMI or modifications of glucose and lipid biochemical parameters.

## 1. Introduction

Obesity is a multifactorial chronic disease defined by excess adipose mass and adipose tissue expansion, which occur through adipocyte hypertrophy and hyperplasia. There is increasing evidence that adipose tissue participates in several metabolic activities and is involved in lipoprotein metabolism [1,2,3,4]. In overweight/obese subjects, the changes in the morphology and function of adipose tissue are associated with alterations of plasma high-density lipoprotein cholesterol (HDL-C) levels and HDL function. Low HDL-C levels [5] and dysfunctional HDL with lower antioxidant and anti-inflammatory properties have been frequently observed in obese patients [6,7]. In detail, HDLs isolated from obese subjects exhibit lower levels and activity of the enzyme paraoxonase-1 (PON1) [8,9,10,11]. PON1 is a 43–45 kDa glycoprotein, mainly synthesized by the liver, that circulates associated at the HDL surface and contributes to the anti-atherogenic and anti-inflammatory properties of this lipoprotein class [12,13,14]. Different mechanisms contribute to the anti-atherogenic properties of PON1; the enzyme protects low-density lipoproteins (LDLs) against oxidative stress, reduces macrophage foam cell formation, reduces the ability of macrophages to uptake ox-LDLs and inhibits the synthesis of monocyte chemotactic protein 1 (MCP-1). Therefore, a decrease in PON1 activity is associated with impaired HDL functions [9,11].

Abdominal adiposity is also associated with a state of low-grade systemic inflammation; adipose tissue infiltrating macrophages and neutrophils contributes to the increased secretion of pro-inflammatory cytokines (i.e., TNF-α) and pro-inflammatory and pro-oxidant proteins such as myeloperoxidase (MPO) [2]. Higher levels of MPO are reported in serums of obese subjects [15,16]. In addition, serum levels of MPO correlate with BMI and are associated with increased markers of inflammation and insulin resistance [15]. The enzyme MPO is one of the main factors involved in the oxidative stress of plasma lipoproteins. In fact, MPO generates hypochlorous acid, which oxidatively damages HDL lipids and apoproteins, including the enzyme PON1 that is associated with HDL surfaces. The compositional changes due to MPO impair the antioxidant properties and the reverse cholesterol transport (RCT) ability of the HDL [17]. A link between MPO and PON1 on the HDL has been described, as they reciprocally regulate each other’s activity. PON1 exerts a protective role against the lipid peroxidation carried out by MPO; on the contrary, MPO promotes site-specific oxidative modification, which can lead to the impairment of PON1 activity [18,19,20]. Therefore, an increase in the serum MPO/PON1 ratio is considered a potential indicator of dysfunctional HDL [18,21]. In addition, pro-oxidant molecules generated by MPO also increase the conversion of LDL into ox-LDL [18,19,20].

Dysfunctional lipoproteins and oxidative stress are involved in the development of several complications and metabolic disorders associated with obesity, including cardiovascular complications, type 2 diabetes, cancer and hepatic and renal dysfunction [13,22,23,24,25], so they represent a possible therapeutic target.

Several meta-analyses have demonstrated evidence that physical activity exerts a key role in the prevention of dysmetabolic diseases and decreases fat around the waist and total body fat, slowing the development of abdominal obesity [26,27]. In addition, positive effects have been described on lipoprotein levels. In sedentary obesity, increased physical activity is generally associated with an increase in HDL cholesterol and a decrease in triglycerides, whereas the results on LDL cholesterol are inconsistent [28,29]. Physical exercise, apart from inducing quantitative alterations in serum lipids, exerts a beneficial impact on HDL particle maturation, composition and functions [30,31]. The impact of exercise on HDL function depends on several factors, including exercise type, intensity and duration, as well as the characteristics (age, ethnicity, body mass, baseline HDL levels, diet, medications, etc.) of subjects enrolled [30,32].

To further study the effect of physical activity on HDL functional properties and oxidative stress in overweight and obese subjects, the effects of a training intervention protocol consisting of seven weeks, including combined resistance and conditioning training, were investigated. Anthropometric parameters were studied in all subjects. Moreover, the activity of the antioxidant enzyme PON1 and the redox activity of HDLs were evaluated in the subjects before and after the training intervention. MPO levels and biochemical markers of oxidative stress in the serums of the subjects (ox-LDL and total antioxidant capacity) were also studied.

## 2. Materials and Methods

### 2.1. Subjects

Recruitment was carried out through announcements on social media and posters on message boards in Umeå, Sweden. The choice of participants was intentionally non-probabilistic, respecting the eligibility criteria summarized in Table 1. The current study included men and women between the ages of 20 and 35, with BMIs ranging from 27.0 to 35.0 kg/m^2^, who had not engaged in regular exercise for at least 12 months prior to the study. Participants were required to be free of any preexisting diagnosed diseases and able to participate in physical training without any injury or medical condition that would restrict their involvement in strength and conditioning exercises. Exclusion criteria were anemia, smoking, pregnancy, blood pressure readings exceeding 140/90 mmHg when attending the laboratory and any health concerns such as upper respiratory tract infections, overload injuries or other medical conditions that may arise during the intervention (Table 1).

A total of 21 subjects fulfilled the inclusion criteria and volunteered to participate in the study. Before inclusion, eligible subjects were given written and oral information about the study and the possible risks associated with physical tests, training and blood collection (which were assessed as small). All volunteers visited the orthopedic clinic at Norrlands University Hospital, Umeå, Sweden, to have a medical assessment completed by an experienced physician. The medical assessment included questions regarding medical history, existing medical conditions, allergies, medications and family medical history. Additionally, blood pressure, heart rate, breathing, eyes, throat, lymph nodes, reflexes, joints and muscles were checked. No medical disorders or symptoms were found in any person. Subjects included in the study did not receive any type of dietary intervention and were instructed to maintain their dietary habits and routine activities. All included subjects provided their written and informed consent for participation. The experimental study protocol was conducted in accordance with the Helsinki Declaration and approved (Dnr.13/262-31) by the Regional Ethical Review Board, Umeå, Sweden. Of the 21 included subjects, 18 completed the study; the reasons for withdrawal from the study were personal matters. The sample size (*n* = 18) was based on an expected improvement of 10 percent in VO_2_max with 7 weeks of training, according to the data not earlier investigations, a power of 0.8 and alfa = 0.05.

### 2.2. Exercise Training

**Figure 1:**
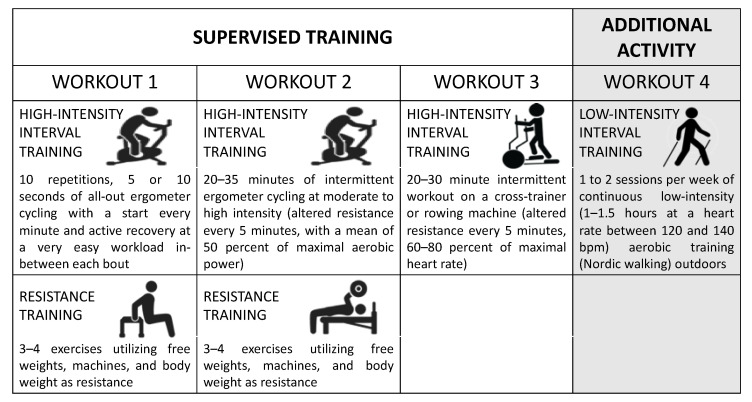
Overview of weekly workouts for overweight/obese subjects. The figure was generated using Microsoft PowerPoint using royalty-free vectors from https://stock.adobe.com/ (accessed on 25 September 2023).

The training intervention protocol consisted of seven weeks, including combined resistance and conditioning training four to five times each week. The same training program was applied to both men and women. Both the endurance and resistance training shifted between easier and heavier sessions, and the intensity progressed gradually over time to improve muscle strength and aerobic functions. The goal was to improve muscle strength and endurance in a large proportion of muscle groups. All participants visited the Sports Medicine facility to attend supervised training three times per week following a 10 min easy warm-up (on a cycle ergometer or cross-trainer). Figure 1 summarizes the weekly workout program. One workout consisted of the following:

1. A total of 10 repetitions of 5 or 10 s (varied from week to week) of all-out ergometer cycling, with a start every minute and active recovery at a very easy workload in between each bout.
2. A total of 20–35 min of intermittent ergometer cycling at moderate-to-high intensity (altered resistance every 5 min, with a mean of 50 per cent of maximal aerobic power).
3. One 20–30 min intermittent workout with a cross-trainer or rowing machine (altered resistance every 5 min, with 60–80 per cent of maximal heart rate).

Resistance exercises (3–4 exercises) were performed in the second halves of No. 1 and No. 2 workouts. The resistance training consisted of exercises with free weights, machines and body weight as resistance. The resistance training sessions shifted focus every second week, focusing on endurance strength (10–30 repetitions per 2–4 sets/exercise) one week and muscle hypertrophy (6–8 repetitions and 3–6 sets/exercise) the other. The resistance was increased if more repetitions were achieved than the upper limit of the determined repetition range. Likewise, the weights were decreased if fewer repetitions than the lower repetition limit were achieved.

In addition to the supervised training, participants also performed one to two sessions per week of continuous low-intensity (1–1.5 h at a heart rate between 120 and 140 bpm) aerobic training (Nordic walking) on their own in an optional outdoor environment. Each walking session was measured with a heart rate monitor with GPS (Polar RS800CX, Polar Electro Oy, Kempele, Finland). The training program is available on request.

### 2.3. Test for Aerobic Capacity and Muscle Strength

All participant performed an incremental cycling test under a cycle ergometer (Monark, 894, Monark Exercise AB, Vansbro, Sweden) under standard environmental conditions; the room temperature was controlled and maintained at 19–20 °C. The settings on the cycle ergometer were adjusted for each individual and reused at the retest after the intervention period. Feet were securely strapped to the pedals. Prior to the test, participants completed a 5 min warm-up at 30–50 watts with a pedaling rate of 60–70 rpm. The submaximal test started at 30 W with continuous increases of 15 W every fourth minute until the 90 W level was completed (pedaling rate of 70 rpm). Each four-minute interval was followed by one minute of recovery sitting on the cycle ergometer before the next interval began. After one minute of recovery following completion of the fifth submaximal workload, the VO_2_max test began at 105 W with an increase of 15 W each minute until exhaustion (VO_2_max). The participants were allowed to choose an optional pedaling rate. The VO_2_max was determined as the highest mean VO_2_ of a cohesive 60 s period. Analyses of air flow and respiratory gases were performed with a Jaeger Oxycon Pro system (Erich Jaeger GmbH, Hochberg, Germany). To assess lower body strength, each participant performed a test to determine three repetition maximum (3 RM) in leg press with a plate-loaded leg press machine (Gymleco, Eskilstuna, Sweden).

### 2.4. Levels of Lipoproteins, Insulin, Glucose and hsCRP

Assessments of body weight and height were completed through standard clinical procedures, and body composition was determined using intelligent dual-energy X-ray absorptiometry technology (iDXA, GE Healthcare, Madison, WI, USA).

Serums were obtained from venous blood samples collected in the morning following an overnight fast, prior to and following 7 weeks of exercise training. The collected venous blood (via a venflon iv catheter) was handled according to the manufacturer’s instructions (BD Vacutainer^®^ blood collection tubes) and centrifuged at 3000× *g* and +4 °C for 10 min then stored in −80 °C until time of analysis. Fasting glucose, high-sensitivity CRP (hsCRP), insulin, blood lipids and lipoproteins were evaluated at the accredited Clinical Chemistry Laboratory, Uppsala University Hospital, Sweden. Glucose, hsCRP, blood lipids and lipoproteins were analyzed on an Architect 8200 (Abbott Laboratories, Abbott Park, IL, USA) with reagents from the same manufacturer. Insulin was measured using a Cobas E analyzer (Roche Diagnostics, Mannheim, Germany). Homeostatic model assessment for insulin resistance (HOMA-IR) and non-HDL cholesterol were calculated as described by Wallace et al. [33] and Ridefelt et al. [34], respectively.

### 2.5. Serum Total Antioxidant Potential (PAT)

Serum total antioxidant potential was measured using oxygen radical absorbance capacity (ORAC) adapted for semi-automated measurement on a 96-well microplate reader (Synergy HT; BioTek, Winooski, VT, USA) [35].

### 2.6. Levels of Oxidized LDL (ox-LDL)

ox-LDL in serum was determined by a sandwich ELISA procedure using the murine monoclonal antibody mAB-4E6 as the capture antibody and a peroxidase-conjugated antibody against oxidized apolipoprotein B (ApoB) bound to the solid phase (Mercodia AB, Uppsala, Sweden). The assay was performed according to the instructions of the manufacturer. Results were reported as U for L of serum. Sensitivity of ox-LDL measurements was <1 mU/L, and intra- and inter-assay coefficients of variation were 1.7–2.7% and 7.8–9.7%, respectively.

### 2.7. Myeloperoxidase (MPO) Levels

A solid-phase two-site MPO ELISA Kit (Mercodia AB, Uppsala, Sweden) was used to evaluate serum MPO. The assay was performed according to the instructions of the manufacturer. Serum samples of controls and patients were included on each plate. Results were reported as ng of MPO per mL of serum. Sensitivity of ox-MPO measurements was ≤3 ng/mL, and intra- and inter-assay coefficients of variation were 3.0–4.4% and 5.5–9.9%, respectively.

### 2.8. Paraoxonase-1 (PON1) Activity

PON1 activity was assayed in serum by using three substrates: phenyl acetate, paraoxon or dihydrocoumarin. All assays of PON1 activity were performed in a 96-well plate, in a total reaction volume of 200 µL. Each 96-well plate included blank samples to monitor spontaneous hydrolysis of substrates [36].

Paraoxonase (PON) Activity. The basal assay mixture included 50 mM glycine/NaOH with a pH of 10.5, 1 mM CaCl_2_ and 2.0 mmol/L paraoxon. A total of 5 µL of undiluted serum was taken for a total reaction volume of 200 µL. Paraoxon hydrolysis was spectrophotometrically monitored for 8 min (every 15 s) at 412 nm. One unit of PON1 paraoxonase activity was equivalent to one nmol of paraoxon hydrolyzed/min/mL.

Arylesterase (ARE) Activity. Serum samples were diluted 1:10 with 50 mM Tris-HCl with a of pH 8.0 and 1 mM CaCl_2_, and then 5 µL was taken for a total reaction volume of 200 µL. After addition of the substrate phenyl acetate (1 mmol/L), the hydrolysis was monitored at 270 nm for 3 min (every 15 s). One unit of arylesterase activity was equivalent to one µmol of phenyl acetate hydrolyzed/min/mL.

Lactonase (LAC) Activity. Serum samples were diluted 1:10 with 50 mM Tris-HCl with a pH of 7.5 and 1 mM CaCl_2_, and 3 µL was then taken for the assay. After addition of the substrate dihydrocoumarin (DHC) (1.0 mM), the hydrolysis was monitored at 270 nm for 10 min (every 15 s). One unit of lactonase activity was equivalent to one µmol of DHC hydrolyzed/min/mL.

### 2.9. HDL Redox Activity

HDL redox activity was assessed using a fluorometric biochemical cell-free assay [37]. This assay measures the effects of HDL on the rate of oxidation of the fluorogenic probe dihydrorhodamine 123 (DHR). HDLs were isolated from the serum of subjects using selective precipitation with polyethylene glycol. A stock solution of DHR (50 mM) was diluted at 1:1000 in iron-free N-2-hydroxyethylpiperazine-N-2-ethanesulfonic acid-buffered saline (HBS; HEPES 20 mM, NaCl 150 mM, pH 7.4) prepared as previously described [37]. In a 96-well plate, aliquots of HDL (1.25 μg HDL-cholesterol) and DHR working solution (final concentration of 7 μM) were added, and the volume was diluted to 200 μL with HBS buffer. Immediately following DHR addition, the plate was protected from light and placed in a fluorescence plate reader. The fluorescence of each well was assessed at 2 min intervals for 1 h with a microplate reader (Synergy HT, BioTek, Winooski, VT, USA) using a 485/538 nm excitation/emission filter. The oxidation rate was calculated for each well as the slope for the linear regression of fluorescence intensity between 10 and 60 min (DHR oxidation rate or DOR) and expressed as fluorescence units per minute (FU/min). The value was calculated as the mean of quadruplicates for the wells containing only DHR and for samples containing DHR and individual samples.

### 2.10. Statistical Analysis

Statistical analysis was performed with GraphPad Prism (GraphPad, San Diego, CA, USA). All data are expressed as means ± SD. To analyze the difference between pre-intervention and post-intervention values, the Wilcoxon test was used due to the small sample size. To verify the correlations between the clinical and biochemical parameters, the Spearman’s correlation test was used. A *p* value of ≤0.05 was considered statistically significant. Correlation matrix was obtained using Displayr platform for survey data analysis (https://www.displayr.com, accessed on 20 September 2023).

## 3. Results

### 3.1. Anthropometric and Biochemical Characteristics

The seven weeks with combined endurance and resistance training resulted in a significant improvement in VO_2_max as well as muscle strength in parallel to a significant decrease in fat mass measures and significant increase in lean mass, without any significant change in body mass index (BMI) (Table 2). Table 2 also summarizes the blood biochemical parameters of subjects before and after the exercise training. All subjects were metabolically healthy at the beginning of the study, as shown by the levels of fasted glucose, insulin and lipoproteins. In fact, all the participants had lipid parameters within the National Cholesterol Education Program (NCEP) reference ranges [38]. After short-term exercise training, a significant decrease in total cholesterol and fasting insulin was observed (Table 2). No significant changes were observed in the levels of other plasma lipids, C-reactive protein (hsCRP), glucose or HOMA-IR (Table 2). No significant differences were observed in HDL cholesterol and triglyceride levels and other biochemical parameters between men and women enrolled in our study before and after exercise training.

### 3.2. Serum PON1 Activity and MPO Levels

**Figure 2:**
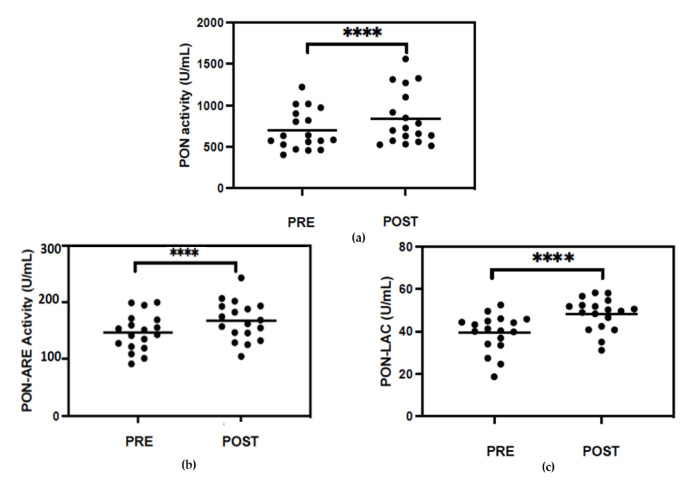
Paraoxonase (PON) (**a**), arylesterase (ARE) (**b**) and lactonase (LAC) (**c**) activities of PON1 in serums of overweight/obese subjects before (PRE) and at the end of the seven-week exercise training (POST). Statistical analysis was performed using Wilcoxon test. **** *p* < 0.0001.

At baseline, PON1-paraoxonase activity showed great variability (403–1220 U/mL) in the subjects included in the study. Higher PON1-paraoxonase activity was observed in all subjects after exercise training (Figure 2a). The increase was statistically significant (*p* < 0.0001) and ranged between 2% and 45%. Similar results were obtained for PON1 arylesterase and lactonase activities (Figure 2b,c) (*p* < 0.0001). PON1 is associated with HDL; therefore, we calculated the standardized enzyme activity value for HDL-C (PON1/HDL-C). There was also significant increase in the ratio of PON1/HDL-C after physical training 

**Figure 3:**
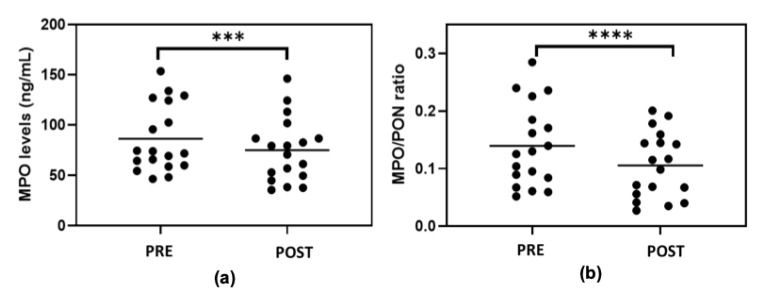
Myeloperoxidase (MPO) levels (**a**) and MPO/PON ratio (**b**) in serums of subjects before (PRE) and at the end of the seven-week exercise training (POST). Statistical analysis was performed using Wilcoxon test. *** *p* < 0.001; **** *p* < 0.0001.

The MPO enzyme levels evaluated in the serums of the subjects ranged from 46 ng/mL to 153 ng/mL. Lower MPO levels were observed in the subjects after exercise training (*p* < 0.001) (Figure 3a); the decrease ranged from −4% to −36%.

The comparison of the ratio between serum MPO level/PON1 paraoxonase activity (MPO/PON1 ratio) showed a decrease in obese subjects after exercise training (Figure 3b) (*p* < 0.0001). Recently, the MPO/PON1 ratio has been suggested as a novel biomarker of HDL functionality [39]; therefore, our data confirm that, although pre- and post-HDL-C levels did not undergo changes, HDL function appeared to be improved following exercise training.

### 3.3. HDL Redox Activity

**Figure 4:**
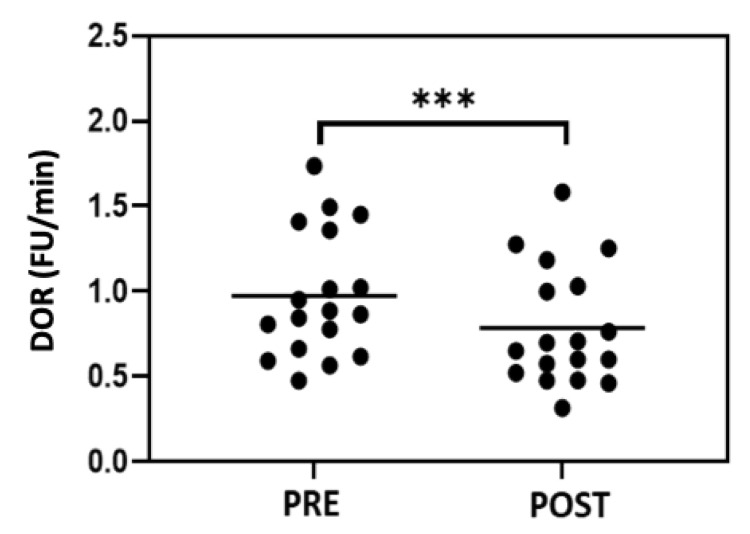
Redox activity of HDLs isolated from serums of subjects before (PRE) and at the end of the seven-week exercise training (POST). Values are reported as oxidation rate of DHR (DOR). Statistical analysis was performed using Wilcoxon test. *** *p* < 0.001.

HDL redox activity was assessed by measuring the increasing fluorescence of dihydrorhodamine 123 oxidation over time. As reported in Figure 4, the mean values of the oxidation rate of DHR (DOR) in HDLs isolated from the subjects were significantly lower after exercise training (*p* < 0.001). These results demonstrate that HDLs from subjects after training have a lower intrinsic ability to be oxidized.

### 3.4. Serum Lipid Peroxidation and Antioxidant Potential

**Figure 5:**
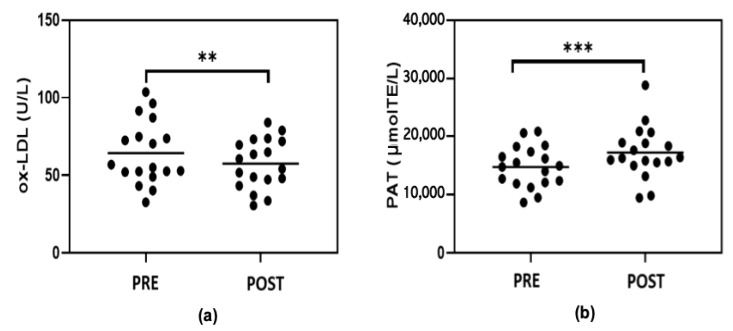
Levels of oxidized LDL (ox-LDL) (**a**) and total antioxidant potential (**b**) of serums of subjects before (PRE) and at the end of the seven-week exercise training (POST). Statistical analysis was performed using Wilcoxon test. ** *p* < 0.05; *** *p* < 0.001.

The levels of oxidized LDL (ox-LDL) in subjects included in the study ranged from 40 U/L to 103 U/L. Exercise training was associated with a significant decrease in ox-LDL levels (*p* < 0.05) (Figure 5a). These data suggest a decrease in lipoprotein oxidation in obese subjects following treatment. The evaluation of the total antioxidant potential (PAT) of the serums (evaluated using ORAC assay) demonstrated an increase in antioxidant capacities following the exercise treatment (Figure 5b).

### 3.5. Correlations

**Figure 6:**
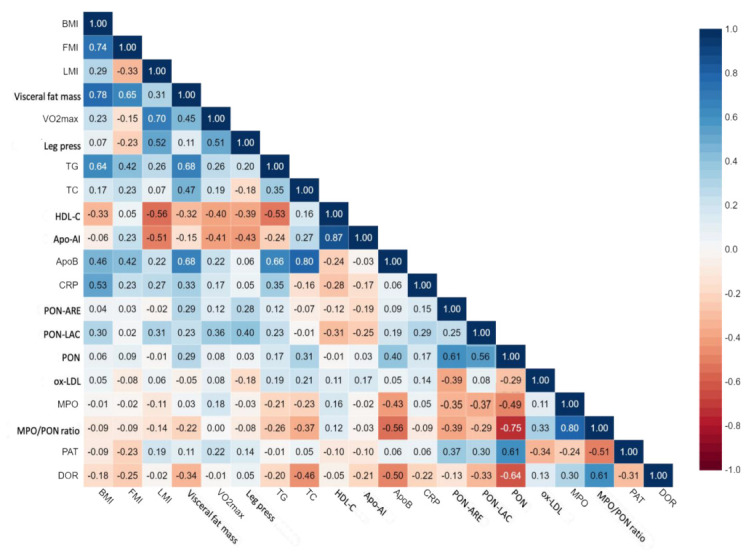
Correlation matrix for clinical and biochemical parameters. Values inside each box represent Spearman’s correlation coefficient. BMI, body mass index; FMI, fat mass index; LMI, lean mass index; VO_2_max, maximal oxygen uptake; TGs, triglycerides; TC, total cholesterol; HDL-C, high-density lipoprotein cholesterol; CRP, C-reactive protein; PON-ARE, PON1-arylestrase activity; PON-LAC, PON1 lactonase activity; PON, PON1 paraoxonase activity; ox-LDL, oxidized LDL; MPO, myeloperoxidase; PAT, serum total antioxidant potential; DOR oxidation rate of DHR.

The pairwise correlation between each of the studied clinical and biochemical parameters, which were evaluated in the subjects before and at the end of seven-week training, was considerable (Figure 6). The LMI was significantly positively correlated to the VO_2_max (r = 0.61, *n* = 36, *p* < 0.001); these results confirm that subjects with a larger muscle mass show higher oxygen consumption. A significant positive correlation was observed between the BMI of subjects and their CRP (r = 0.53, *n* = 36, *p* < 0.002) and TG (r = 0.64, *n* = 36, *p* < 0.0001) and ApoB (r = 0.46, *n* = 36, *p* < 0.001) plasma levels (Figure 6). A significant positive correlation was also established between the visceral fat mass of the subjects and their TG (r = 0.68, *n* = 36, *p* < 0.0001), TC (r = 0.47, *n* = 36, *p* < 0.005) and ApoB plasma levels (r = 0.68, *n* = 36, *p* < 0.0001); weak negative correlations between visceral fat mass and serum HDL-C levels (r = −0.32, *n* = 36, *p* < 0.05) and HDL redox activity (r = −0.34, *n* = 36, *p* < 0.05) were observed (Figure 6).

Serum PON1 activities were negatively correlated with MPO levels (Figure 5). A significant positive correlation was established between HDL redox activity and the MPO/PON1 ratio (r = 0.61, *n* = 36, *p* < 0.0001) and PON1 paraoxonase activity (r = −0.64, *n* = 36, *p* < 0.0001) in the serums of obese subjects before and after training. Furthermore, MPO/PON1 ratio values were negatively correlated with PAT values (r = −0.51, *n* = 36, *p* < 0.003) and, albeit not as strongly, positively correlated to ox-LDL levels (r = 0.33, *n* = 36, *p* < 0.05) observed in the serums of obese subjects before and after training.

## 4. Discussion

Previous studies have investigated the effects of physical activity on HDL functions in overweight and normal-weight subjects, as summarized in recent metanalyses [30,32]. Nevertheless, when dealing with the effects of combined physical exercise (aerobic and resistance training), evidence is still scarce for the population with obesity. Combined physical exercise has been appointed as an ideal treatment for overweight and obese people, due to its concomitant effects on cardiorespiratory and muscular fitness, as recommended by the American College of Sports Medicine [40]. Furthermore, a recent meta-analysis of randomized clinical trials showed that exercise training increased HDL-C to a greater extent than dietary intervention alone for overweight individuals [28,41]. In our study, marked improvements in VO_2_max and muscle strength were observed after the training period. Significant reductions in total and visceral adipose tissue mass and waist circumference were observed without any significant decrease in body weight and BMI. These data are in agreement with other studies [42]. A significant increase in the activity of the antioxidant HDL-associated enzyme paraoxonase-1 (PON1) and a decrease in MPO levels, with a decrease in the MPO/PON1 ratio, were observed in the serums of obese subjects after a 7-week training intervention. Moreover, HDLs isolated from the serums of obese subjects showed a lower susceptibility to oxidative stress and higher antioxidant activity. The improvement of HDL functionality after exercise training was associated with a significant decrease in levels of ox-LDL and an increase in serum antioxidant potential. These changes occurred despite no significant change in HDL cholesterol levels or modifications of other plasma lipids. These results confirm that lifestyle changes, such as physical exercise, improved HDL function even before there were appreciable changes in HDL levels [30,32,43].

**Figure 7:**
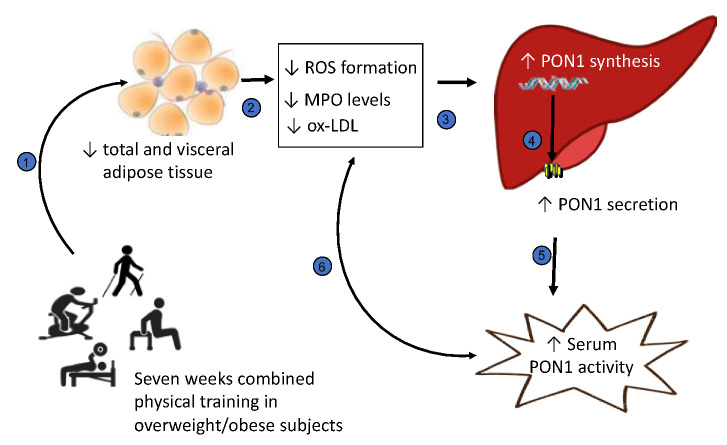
Effect of exercise training on paraoxonase-1 (PON1) activity in overweight/obese subjects. Exercise training is associated with a reduction in total and visceral adipose tissue (1). There is a consequent decrease in MPO levels and markers of lipid peroxidation in blood (2). In hepatic cells, the decreases in lipid peroxidation and pro-inflammatory products could modulate PON1 gene expression (3). The increased PON1 secretion (4) results in higher PON1 activity in serum (5). The higher PON1 activity could be also due to the reduced inhibitory effect exerted by lipid peroxidation products and by MPO; on the other hand, the higher PON1 activity could contribute to reduce ox-LDL and MPO pro-oxidant activity (6) (↑ increase; ↓ decrease). The figure was generated using Microsoft PowerPoint (version 16.16.27) using royalty-free vectors from https://freesvg.org (accessed on 19 August 2023) and https://stock.adobe.com/ (accessed on 25 September 2023).

The PON1 enzyme has been reported to contribute to the anti-atherogenic and anti-inflammatory properties of HDL [12,14]. In obese subjects, the decrease in PON1 activity is associated with a loss of ability to inhibit lipoprotein oxidation and correlated with increased oxidative stress [9,11]. The increase in PON1 activities, including the lactonase activity, the native enzyme activity [44], after a 7-week training intervention supports the positive effect of physical activity on HDL. Previous studies have shown a significant improvement in PON1 activity and HDL functionality after a period of physical exercise practice in normal-weight or obese subjects [45,46,47,48,49]. However, contrasting results have been reported by other authors, and no significant modification in PON1 activity and HDL function have been observed after physical activity in overweight/obese subjects [50,51]. These data suggest that the impact of exercise on HDL function and PON1 activity depends on several factors, including exercise type, intensity and duration, as well as clinical characteristics (age, ethnicity, body mass, diet, medications, etc.) [30,32,52]. The molecular mechanisms involved in the increased PON1 activities after exercise training may include an effect on PON1 hepatic synthesis or secretion and/or modification of PON1 interactions with HDL. Previous studies have reported that high ROS and pro-inflammatory cytokines (TNF-a, IL-1 and IL-6) from the adipose tissue and liver modulate hepatic PON1 synthesis through cell receptor-mediated signaling pathways and nuclear receptors (such as peroxisome proliferator-activated receptors, PPARs) [53,54]. Moreover, PON1 was found to be inactivated by oxidized lipids [55]. We suggest that physical exercise, associated with a significant reduction in total and visceral adipose tissue mass, results in reduced oxidative stress and pro-inflammatory and pro-oxidant proteins, such as MPO, leading to the increase in PON1 expression and activity in overweight/obese subjects (Figure 7). The significant negative correlation observed between PON1 activity and MPO levels of the subjects included in our study confirm the key role of MPO in modulating PON1 activity. PON1 and MPO are both associated with the HDL lipid surface, forming a ternary complex [56]. HDL is a selective in vivo target for MPO-catalyzed oxidation [57]. MPO generates hypochlorous acid, which oxidatively damages lipid and protein HDLs. Extensive MPO-mediated posttranslational modifications, including the oxidation of tryptophan, tyrosine and methionine residues, as well as the carbamylation of lysine residues, are detected in ApoAI and PON1. These modifications are associated with impaired antioxidative and RCT abilities of HDL and also with an increased conversion of LDL into ox-LDL [18,19,20,57].

Previous studies have investigated the modifications of MPO levels after dietary intervention and aerobic exercise in obese subjects [51] and subjects with metabolic syndrome (MetS) [43,58,59]. A decrease in serum MPO levels was shown after 3 weeks of a low-fat diet and aerobic exercise in 31 obese men, 15 of whom had MetS; the modifications were associated with decreases in BMI, serum lipids and markers of lipid peroxidation (8-isoprostaglandin F2α) and markers of inflammation such as CRP, soluble ICAM-1, soluble P-selectin and matrix metalloproteinase-9 [51]. Mathew et al. reported a significant decrease in the MPO products 3-chlorotyrosine and 3-nitrotyrosine in HDL after 12 weeks of moderate exercise and dietary changes in patients with MetS, even in the absence of modification of body weight; the decrease in these products was correlated with improvement in HDL cholesterol efflux capacity [43]. Nonetheless, to the current researchers’ knowledge, no studies have yet been conducted to investigate the effects of physical activity, without dietary intervention, on MPO levels and on the MPO/PON1 ratio in obesity. Our results showed a significant negative correlation between serum MPO/PON ratios and HDL redox activity. Therefore, our study confirms that the combined use of these two important HDL-associated enzymes, PON1 and MPO, and the evaluation of the MPO/PON1 ratio are valuable markers of HDL functionality. The improvement of HDL functionality after exercise training was associated with a significant decrease in the levels of ox-LDL and an increase in serum antioxidant capacity in the serums of overweight/obese subjects. All these results suggest that overweight/obese adults may benefit from combined physical exercise programs in many metabolic aspects that are related to protection against the development of cardiovascular disease.

## 5. Conclusions

Obesity represents a significant public health concern with one third of adults classified as living with obesity. We found that in overweight/obese subjects, the combined physical exercise program, without active modifications of dietary habits, improved aerobic capacity and muscle strength and decreased total and visceral adipose tissue mass and waist circumference without any significant decrease in body weight. These modifications were associated with a reduced MPO/PON1 ratio and increased HDL functionality, along with an improvement of the oxidant/antioxidant balance. These dynamic changes in the HDL function and oxidation state of subjects in short-term combined physical exercise programs prior to changes in HDL cholesterol levels and without modifications of the dietary habits or BMIs of the subjects, are a key finding of this work. These data indicate that HDL functional modifications are realized early and are independent of HDL cholesterol improvement and modification of body weight. It has to be stressed that the subjects included in our study were without any alteration of metabolic parameters associated with cardiovascular diseases. In fact, all the participants had lipid parameters within the National Cholesterol Education Program (NCEP) reference ranges.

Some limitations of the current study must be considered, including the small number of obese subjects included and the absence of a control group of overweight/obese subjects who did not receive any type of training intervention.

These results could contribute to the whole of solid evidence that can guide the prescription of physical exercise treatment for overweight/obese subjects, aiming to reduce cardiovascular risk and improve general health. Moreover, current communication strategies surrounding physical activity for overweight/obese subjects are manly related to dietary therapy and weight loss; however, this approach often appears inadequate. Information about other health-relevant outcomes, rather than weight loss by itself, could make physical activity more relevant and compelling for overweight/obese subjects to initiate and sustain.

## Figures and Tables

**Table 1:** Inclusion and exclusion criteria.

Inclusion Criteria	Exclusion Criteria
⋅ Age between 20 and 35 years ⋅ BMI > 27.0 kg/m^2^ ⋅ No regular exercise for at least 12 months prior to the study ⋅ No injury or medical condition that restricts participation in the exercises	⋅ Smoking ⋅ Blood pressure > 140/90 mmHg ⋅ Anemia (Hb < 120 g/L women, <130 g/L men) ⋅ Pregnancy ⋅ Preexisting CVD, diabetes or any other diagnosed disease ⋅ Refusal to give informed consent ⋅ Withdrawal from the intervention for personal reasons

**Table 2:** Anthropometric and biochemical characteristics of subjects before (PRE) and at the end (POST) of the seven-week exercise training.

	PRE (Mean ± SD)	POST (Mean ± SD)	*p* Value
Anthropometrics			
Body weight (kg)	93.2 ± 12.5	93.3 ± 12.4	0.990
BMI (kg‧m^−2^)	30.3 ± 2.5	30.3 ± 2.6	0.867
Fat mass (kg)	37.1 ± 8.2	35.7 ± 8.3	<0.001
FMI (kg‧m^−2^)	12.1 ± 2.6	11.6 ± 2.6	<0.001
Lean mass (kg)	52.3 ± 8.6	54.4 ± 8.6	<0.0001
LMI (kg‧m^−2^)	17.2 ± 1.6	17.6 ± 1.7	0.0002
Visceral fat mass (kg)	1.1 ± 0.6	0.9 ± 0.8	0.0076
Waist circumference (cm)	95.6 ± 8.6	93.8 ± 8.3	0.0081
Aerobic capacity and muscle strength			
VO_2_max (L‧min^−1^)	3.3 ± 0.7	3.7 ± 0.8	0.0002
Leg press (kg)	280.0 ± 86.7	483.3 ± 177.4	<0.0001
Biochemical parameters			
TGs (mmol/L)	1.38 ± 0.72	1.39 ± 0.77	0.89
TC (mmol/L)	5.07 ± 0.99	4.61 ± 1.48	0.031
HDL-C (mmol/L)	1.54 ± 0.29	1.47 ± 0.32	0.123
Non-HDL cholesterol (mmol/L)	3.53 ± 1.01	3.14 ± 1.45	0.059
Apo AI (g/L)	1.62 ± 0.22	1.56 ± 0.19	0.44
Apo B (g/L)	0.92 ± 0.26	0.90 ± 0.31	0.66
hsCRP (mg/L)	3.39 ± 3,51	2.59 ± 2.17	0.348
HOMA-IR	3.72 ± 2,09	2.99 ± 1.93	0.106
Fasting glucose (mmol/L)	5.27 ± 0.46	5.52 ± 0.56	0.189
Fasting insulin (mUI/L)	16.40 ± 11.36	11.84 ± 6.56	0.036

Data are means ± S.D. Statistical analysis was performed using Wilcoxon test. BMI, body mass index; FMI, fat mass index; LMI, lean mass index; BMI, body mass index; VO_2_max, maximal oxygen uptake; TGs, triglycerides; TC, total cholesterol; HDL-C, high-density lipoprotein cholesterol; Apo AI, Apolipoprotein AI; Apo B, Apolipoprotein B; hsCRP, C-reactive protein; HOMA-IR, homeostatic model assessment of insulin resistance.

## Data Availability

The data presented in this study are available on request from the corresponding author. The data are not publicly available due to ethical restrictions.

## References

[1] Zhang T., Chen J., Tang X., Luo Q., Xu D., Yu B. (2019). Interaction between adipocytes and high-density lipoprotein:new insights into the mechanism of obesity-induced dyslipidemia and atherosclerosis. Lipids Health Dis..

[2] Kawai T., Autieri M.V., Scalia R. (2021). Adipose tissue inflammation and metabolic dysfunction in obesity. Am. J. Physiol. Cell Physiol..

[3] Luo L., Liu M. (2016). Adipose tissue in control of metabolism. J. Endocrinol..

[4] Reho J.J., Rahmouni K. (2017). Oxidative and inflammatory signals in obesity-associated vascular abnormalities. Clin. Sci..

[5] Wang H., Peng D.Q. (2011). New insights into the mechanism of low high-density lipoprotein cholesterol in obesity. Lipids Health Dis..

[6] Stadler J.T., Lackner S., Morkl S., Trakaki A., Scharnagl H., Borenich A., Wonisch W., Mangge H., Zelzer S., Meier-Allard N. (2021). Obesity Affects HDL Metabolism, Composition and Subclass Distribution. Biomedicines.

[7] Stadler J.T., Marsche G. (2020). Obesity-Related Changes in High-Density Lipoprotein Metabolism and Function. Int. J. Mol. Sci..

[8] Azmi S., Ferdousi M., Liu Y., Adam S., Siahmansur T., Ponirakis G., Marshall A., Petropoulos I.N., Ho J.H., Syed A.A. (2021). The role of abnormalities of lipoproteins and HDL functionality in small fibre dysfunction in people with severe obesity. Sci. Rep..

[9] Ferretti G., Bacchetti T., Masciangelo S., Bicchiega V. (2010). HDL-paraoxonase and membrane lipid peroxidation: A comparison between healthy and obese subjects. Obesity.

[10] Dogan K., Senes M., Karaca A., Kayalp D., Kan S., Gulcelik N.E., Aral Y., Yucel D. (2021). HDL subgroups and their paraoxonase-1 activity in the obese, overweight and normal weight subjects. Int. J. Clin. Pract..

[11] Ferretti G., Bacchetti T., Masciangelo S., Grugni G., Bicchiega V. (2012). Altered inflammation, paraoxonase-1 activity and HDL physicochemical properties in obese humans with and without Prader-Willi syndrome. Dis. Model. Mech..

[12] Khalil A., Kamtchueng Simo O., Ikhlef S., Berrougui H. (2017). The role of paraoxonase 1 in regulating high-density lipoprotein functionality during aging. Can. J. Physiol. Pharmacol..

[13] Bacchetti T., Ferretti G., Sahebkar A. (2019). The role of paraoxonase in cancer. Semin. Cancer Biol..

[14] Kotur-Stevuljevic J., Vekic J., Stefanovic A., Zeljkovic A., Ninic A., Ivanisevic J., Miljkovic M., Sopic M., Munjas J., Mihajlovic M. (2020). Paraoxonase 1 and atherosclerosis-related diseases. Biofactors.

[15] Olza J., Aguilera C.M., Gil-Campos M., Leis R., Bueno G., Martinez-Jimenez M.D., Valle M., Canete R., Tojo R., Moreno L.A. (2012). Myeloperoxidase is an early biomarker of inflammation and cardiovascular risk in prepubertal obese children. Diabetes Care.

[16] Qaddoumi M.G., Alanbaei M., Hammad M.M., Al Khairi I., Cherian P., Channanath A., Thanaraj T.A., Al-Mulla F., Abu-Farha M., Abubaker J. (2020). Investigating the Role of Myeloperoxidase and Angiopoietin-like Protein 6 in Obesity and Diabetes. Sci. Rep..

[17] Bergt C., Pennathur S., Fu X., Byun J., O’Brien K., McDonald T.O., Singh P., Anantharamaiah G.M., Chait A., Brunzell J. (2004). The myeloperoxidase product hypochlorous acid oxidizes HDL in the human artery wall and impairs ABCA1-dependent cholesterol transport. Proc. Natl. Acad. Sci. USA.

[18] Bacchetti T., Ferretti G., Carbone F., Ministrini S., Montecucco F., Jamialahmadi T., Sahebkar A. (2021). Dysfunctional High-density Lipoprotein: The Role of Myeloperoxidase and Paraoxonase-1. Curr. Med. Chem..

[19] Marsche G., Stadler J.T., Kargl J., Holzer M. (2022). Understanding Myeloperoxidase-Induced Damage to HDL Structure and Function in the Vessel Wall: Implications for HDL-Based Therapies. Antioxidants.

[20] Jin Z., Zhou L., Tian R., Lu N. (2021). Myeloperoxidase Targets Apolipoprotein A-I for Site-Specific Tyrosine Chlorination in Atherosclerotic Lesions and Generates Dysfunctional High-Density Lipoprotein. Chem. Res. Toxicol..

[21] Haraguchi Y., Toh R., Hasokawa M., Nakajima H., Honjo T., Otsui K., Mori K., Miyamoto-Sasaki M., Shinohara M., Nishimura K. (2014). Serum myeloperoxidase/paraoxonase 1 ratio as potential indicator of dysfunctional high-density lipoprotein and risk stratification in coronary artery disease. Atherosclerosis.

[22] Ertek S. (2018). High-density Lipoprotein (HDL) Dysfunction and the Future of HDL. Curr. Vasc. Pharmacol..

[23] Zvintzou E., Xepapadaki E., Skroubis G., Mparnia V., Giannatou K., Benabdellah K., Kypreos K.E. (2023). High-Density Lipoprotein in Metabolic Disorders and Beyond: An Exciting New World Full of Challenges and Opportunities. Pharmaceuticals.

[24] Tsompanidi E.M., Brinkmeier M.S., Fotiadou E.H., Giakoumi S.M., Kypreos K.E. (2010). HDL biogenesis and functions: Role of HDL quality and quantity in atherosclerosis. Atherosclerosis.

[25] Cho K.H. (2022). The Current Status of Research on High-Density Lipoproteins (HDL): A Paradigm Shift from HDL Quantity to HDL Quality and HDL Functionality. Int. J. Mol. Sci..

[26] Chang Y.H., Yang H.Y., Shun S.C. (2021). Effect of exercise intervention dosage on reducing visceral adipose tissue: A systematic review and network meta-analysis of randomized controlled trials. Int. J. Obes..

[27] Rao S., Pandey A., Garg S., Park B., Mayo H., Despres J.P., Kumbhani D., de Lemos J.A., Neeland I.J. (2019). Effect of Exercise and Pharmacological Interventions on Visceral Adiposity: A Systematic Review and Meta-analysis of Long-term Randomized Controlled Trials. Mayo Clin. Proc..

[28] Khalafi M., Sakhaei M.H., Kazeminasab F., Rosenkranz S.K., Symonds M.E. (2023). Exercise training, dietary intervention, or combined interventions and their effects on lipid profiles in adults with overweight and obesity: A systematic review and meta-analysis of randomized clinical trials. Nutr. Metab. Cardiovasc. Dis..

[29] Jamka M., Makarewicz-Bukowska A., Bokayeva K., Smidowicz A., Geltz J., Kokot M., Kaczmarek N., Zok A., Kononets V., Cielecka-Piontek J. (2022). Comparison of the Effect of Endurance, Strength and Endurance-Strength Training on Glucose and Insulin Homeostasis and the Lipid Profile of Overweight and Obese Subjects: A Systematic Review and Meta-Analysis. Int. J. Environ. Res. Public Health.

[30] Franczyk B., Gluba-Brzozka A., Cialkowska-Rysz A., Lawinski J., Rysz J. (2023). The Impact of Aerobic Exercise on HDL Quantity and Quality: A Narrative Review. Int. J. Mol. Sci..

[31] Cho K.H., Nam H.S., Kang D.J., Zee S., Park M.H. (2023). Enhancement of High-Density Lipoprotein (HDL) Quantity and Quality by Regular and Habitual Exercise in Middle-Aged Women with Improvements in Lipid and Apolipoprotein Profiles: Larger Particle Size and Higher Antioxidant Ability of HDL. Int. J. Mol. Sci..

[32] Ruiz-Ramie J.J., Barber J.L., Sarzynski M.A. (2019). Effects of exercise on HDL functionality. Curr. Opin. Lipidol..

[33] Wallace T.M., Levy J.C., Matthews D.R. (2004). Use and abuse of HOMA modeling. Diabetes Care.

[34] Ridefelt P., Hagstrom E., Svensson M.K., Akerfeldt T., Larsson A. (2019). Age- and sex-specific reference values for non-HDL cholesterol and remnant cholesterol derived from the Nordic Reference Interval Project (NORIP). Scand. J. Clin. Lab. Investig..

[35] Bacchetti T., Tullii D., Masciangelo S., Brugè F., Silvestri S., Orlando P., Tiano L., Ferretti G. (2016). Correlation between plasma levels of carotenoid and oxidized low density lipoproteins: A short human intervention study. Integr. Food Nutr. Metab..

[36] Aviram M., Rosenblat M. (2008). Paraoxonases (PON1, PON2, PON3) analyses in vitro and in vivo in relation to cardiovascular diseases. Methods Mol. Biol..

[37] Kelesidis T., Currier J.S., Huynh D., Meriwether D., Charles-Schoeman C., Reddy S.T., Fogelman A.M., Navab M., Yang O.O. (2011). A biochemical fluorometric method for assessing the oxidative properties of HDL. J. Lipid Res..

[38] (2002). Third Report of the National Cholesterol Education Program (NCEP) Expert Panel on Detection, Evaluation, and Treatment of High Blood Cholesterol in Adults (Adult Treatment Panel III) final report. Circulation.

[39] Variji A., Shokri Y., Fallahpour S., Zargari M., Bagheri B., Abediankenari S., Alizadeh A., Mahrooz A. (2019). The combined utility of myeloperoxidase (MPO) and paraoxonase 1 (PON1) as two important HDL-associated enzymes in coronary artery disease: Which has a stronger predictive role?. Atherosclerosis.

[40] Donnelly J.E., Blair S.N., Jakicic J.M., Manore M.M., Rankin J.W., Smith B.K., American College of Sports M. (2009). American College of Sports Medicine Position Stand. Appropriate physical activity intervention strategies for weight loss and prevention of weight regain for adults. Med. Sci. Sports Exerc..

[41] Greene N.P., Martin S.E., Crouse S.F. (2012). Acute exercise and training alter blood lipid and lipoprotein profiles differently in overweight and obese men and women. Obesity.

[42] Wedell-Neergaard A.S., Eriksen L., Gronbaek M., Pedersen B.K., Krogh-Madsen R., Tolstrup J. (2018). Low fitness is associated with abdominal adiposity and low-grade inflammation independent of BMI. PLoS ONE.

[43] Mathew A.V., Li L., Byun J., Guo Y., Michailidis G., Jaiswal M., Chen Y.E., Pop-Busui R., Pennathur S. (2018). Therapeutic Lifestyle Changes Improve HDL Function by Inhibiting Myeloperoxidase-Mediated Oxidation in Patients With Metabolic Syndrome. Diabetes Care.

[44] Petric B., Kunej T., Bavec A. (2021). A Multi-Omics Analysis of PON1 Lactonase Activity in Relation to Human Health and Disease. OMICS.

[45] Cirilli I., Silvestri S., Marcheggiani F., Olivieri F., Galeazzi R., Antonicelli R., Recchioni R., Marcheselli F., Bacchetti T., Tiano L. (2019). Three Months Monitored Metabolic Fitness Modulates Cardiovascular Risk Factors in Diabetic Patients. Diabetes Metab. J..

[46] Evelson P., Gambino G., Travacio M., Jaita G., Verona J., Maroncelli C., Wikinski R., Llesuy S., Brites F. (2002). Higher antioxidant defences in plasma and low density lipoproteins from rugby players. Eur. J. Clin. Investig..

[47] Goldhammer E., Ben-Sira D., Zaid G., Biniamini Y., Maor I., Lanir A., Sagiv M. (2007). Paraoxonase activity following exercise-based cardiac rehabilitation program. J. Cardiopulm. Rehabil. Prev..

[48] Hematinezhad Touli M., Elmieh A., Hosseinpour A. (2022). The Effect of Six-Week Aerobic Exercise Combined with ease max Incr2Green Tea Consumption on PON1 and VOand Apelin, Blood Pressure, and Blood Lipids Reduction in Young Obese Men. Arch. Razi Inst..

[49] Streb A.R., Braga P.G.S., de Melo R.F., Botelho L.J., Maranhao R.C., Del Duca G.F. (2022). Effects of combined physical exercise on plasma lipid variables, paraoxonase 1 activity, and inflammation parameters in adults with obesity: A randomized clinical trial. J. Endocrinol. Investig..

[50] Woudberg N.J., Mendham A.E., Katz A.A., Goedecke J.H., Lecour S. (2018). Exercise intervention alters HDL subclass distribution and function in obese women. Lipids Health Dis..

[51] Roberts C.K., Ng C., Hama S., Eliseo A.J., Barnard R.J. (2006). Effect of a short-term diet and exercise intervention on inflammatory/anti-inflammatory properties of HDL in overweight/obese men with cardiovascular risk factors. J. Appl. Physiol..

[52] Otocka-Kmiecik A., Orlowska-Majdak M. (2009). The role of genetic (PON1 polymorphism) and environmental factors, especially physical activity, in antioxidant function of paraoxonase. Postep. Hig. Med. Dosw..

[53] Fuhrman B. (2012). Regulation of hepatic paraoxonase-1 expression. J. Lipids.

[54] Ferretti G., Bacchetti T. (2012). Effect of dietary lipids on paraoxonase-1 activity and gene expression. Nutr. Metab. Cardiovasc. Dis..

[55] Aviram M., Rosenblat M., Bisgaier C.L., Newton R.S., Primo-Parmo S.L., La Du B.N. (1998). Paraoxonase inhibits high-density lipoprotein oxidation and preserves its functions. A possible peroxidative role for paraoxonase. J. Clin. Investig..

[56] Huang Y., Wu Z., Riwanto M., Gao S., Levison B.S., Gu X., Fu X., Wagner M.A., Besler C., Gerstenecker G. (2013). Myeloperoxidase, paraoxonase-1, and HDL form a functional ternary complex. J. Clin. Investig..

[57] Malle E., Marsche G., Panzenboeck U., Sattler W. (2006). Myeloperoxidase-mediated oxidation of high-density lipoproteins: Fingerprints of newly recognized potential proatherogenic lipoproteins. Arch. Biochem. Biophys..

[58] Oh E.G., Bang S.Y., Kim S.H., Hyun S.S., Chu S.H., Jeon J.Y., Im J.A., Lee J.E., Lee M.K. (2013). Therapeutic lifestyle modification program reduces plasma levels of the chemokines CRP and MCP-1 in subjects with metabolic syndrome. Biol. Res. Nurs..

[59] Pennathur S., Jaiswal M., Vivekanandan-Giri A., White E.A., Ang L., Raffel D.M., Rubenfire M., Pop-Busui R. (2017). Structured lifestyle intervention in patients with the metabolic syndrome mitigates oxidative stress but fails to improve measures of cardiovascular autonomic neuropathy. J. Diabetes Complicat..

